# Behavior and Performance of Suckling Piglets Provided Three Supplemental Heat Sources

**DOI:** 10.3390/ani10071155

**Published:** 2020-07-07

**Authors:** Yunhui Zhu, Yuzhi Li, Michael Reese, Eric Buchanan, Joel Tallaksen, Lee Johnston

**Affiliations:** West Central Research and Outreach Center, University of Minnesota, Morris, MN 56267, USA; zhu00560@morris.umn.edu (Y.Z.); yuzhili@umn.edu (Y.L.); reesem@umn.edu (M.R.); buch0123@morris.umn.edu (E.B.); tall0007@umn.edu (J.T.)

**Keywords:** behavior, performance, piglets, heat source

## Abstract

**Simple Summary:**

To reduce the carbon footprint in swine production, researchers used heated mats powered by solar-energy to replace heat lamps in farrowing barns. This study evaluated electric-heated mat (EM), water-heated mat (WM), and infrared heat lamp (HL) systems as supplemental heat for piglets from birth to weaning. Data were collected from 42 litters of piglets in three trials, with 14 litters in each treatment group. Postural behaviors were video-recorded and performance data were collected from birth to weaning. Results indicate that piglets spent a similar amount of time on EM as under HL. Growth performance of piglets was comparable among the three heat sources. Taking into consideration cost and ease of installation, electricity use, and durability of the three supplemental heat sources, EM appears to be a better choice to provide supplemental heat to suckling piglets compared to HL and WM.

**Abstract:**

This study compared water-heated mats (WM) and electric-heated mats (EM) with heat lamps (HL) as supplemental heat sources for suckling piglets. Forty-two litters were studied in 3 trials. In all trials, behavior of piglets was video-recorded on day 1, 3, 7, 14, and 21 postpartum. Videos were scan-sampled to register postures (lying and standing) and locations (on or away from mat) to assess piglet use of heat sources. Litter size and weight at birth and weaning, and pre-weaning mortality were recorded. Data were analyzed using Glimmix Procedures of SAS. Piglets spent more time on WM than under HL (67.5% vs. 51.0%, *p* = 0.002). No difference in piglet performance between WM and HL was observed, except mortality tended to be higher in WM (22.9% vs. 8.9%; *p* = 0.06). Piglet performance and use of the heat source were comparable for HL and EM. When comparing WM with EM, piglets provided WM spent more time on the mat compared to those provided EM (21.8% vs. 17.1%; *p* = 0.02). No difference in pre-weaning mortality, litter weight, and individual daily gain was observed between WM and EM group. These results suggest EM and HL were comparable to maintain performance and postural behaviors of piglets.

## 1. Introduction

For newborn piglets, the thermoneutral zone is about 34 °C [1]. However, to ensure thermal comfort of sows [2], the temperature in farrowing rooms is controlled to about 20 °C, which is markedly below the thermoneutral zone for newborn piglets [3]. The immature thermoregulatory ability of piglets makes maintenance of body temperature difficult for piglets and makes them prone to suffer hypothermia. Important secondary effects of hypothermia include starvation, disease, and ultimately death because piglets huddle close to the sow for warmth and are crushed when she moves [4,5]. To reduce hypothermia, localized heat is provided for piglets. At a room temperature of about 20 °C, a 150 W heat lamp lowered pre-weaning piglet mortality from all causes [6]. Under similar room temperature, floor heating (30 °C or 33.5 °C) increased the piglets’ ability to maintain normal body temperature, reduced time to first colostrum intake, and improved pre-weaning survival rate [7,8]. 

Localized supplemental heat for piglets can be categorized into two types: radiant heat (heat lamp), and conductive surface heating (heated floors and heated mats) [9]. Heat lamps are used more widely in the commercial swine industry than heated mats or floors but are a less energy efficient thermal aid [10]. The typical wattage of heat lamps (infrared and incandescent) are 100 to 250 W, while typical heat mats (electric-heated) are 60 to 100 W [11,12,13]. Heat lamps account for more than 50% of the total electricity use in farrow-to-wean commercial farms [14,15]. So, replacing heat lamps with heat mats can reduce electricity use in farrowing barns. 

As consumer’s concerns for environmental impacts of food production systems intensify, their desire for food products with reduced carbon footprint is increasing. One way to reduce the carbon footprint of production systems is to generate electricity consumed by the system using solar power instead of the combustion of fossil fuels. In response to consumers’ demand for low carbon footprint meat, we conducted a research project designed to enhance the use of renewable energy in a swine farrowing operation. In this project, we employed a solar photovoltaic array to generate electricity for operation of supplemental heat sources for suckling piglets. As part of this project we evaluated efficacy of heat mats (electric-heated and water-heated) as supplemental heat for piglets in the farrowing barn. The specific objectives of this study were to assess the usage of heat mats by piglets from birth to weaning compared with heat lamps, and to evaluate effects of heat mats on postural behaviors and performance of piglets. 

## 2. Materials and Methods 

This study was conducted at the University of Minnesota’s West Central Research and Outreach Center (WCROC), Morris, MN, from January 2018 to March 2019. All the experimental protocols were reviewed and approved by the University of Minnesota’s Institutional Animal Care and Use Committee (Protocol # 1704-34744A). 

### 2.1. Animals, Housing, and Management 

For all trials, sows (Landrace × Yorkshire) enrolled were mixed parity, and part of the normal production flow at the Center’s Swine Research Unit. Before moving to farrowing rooms, sows were group-housed (15 sows/pen; 4.0 m^2^/sow) in a straw-bedded hoop gestation barn. Each pen in the gestation barn was equipped with a water fountain and individual feeding stalls. Once daily, sows were fed 2.25 kg of a corn-soybean meal based gestation diet formulated to meet or exceed National Research Council (NRC, 2012) nutrient requirements [16]. Sows had free access to water fountains. 

**Figure 1:**
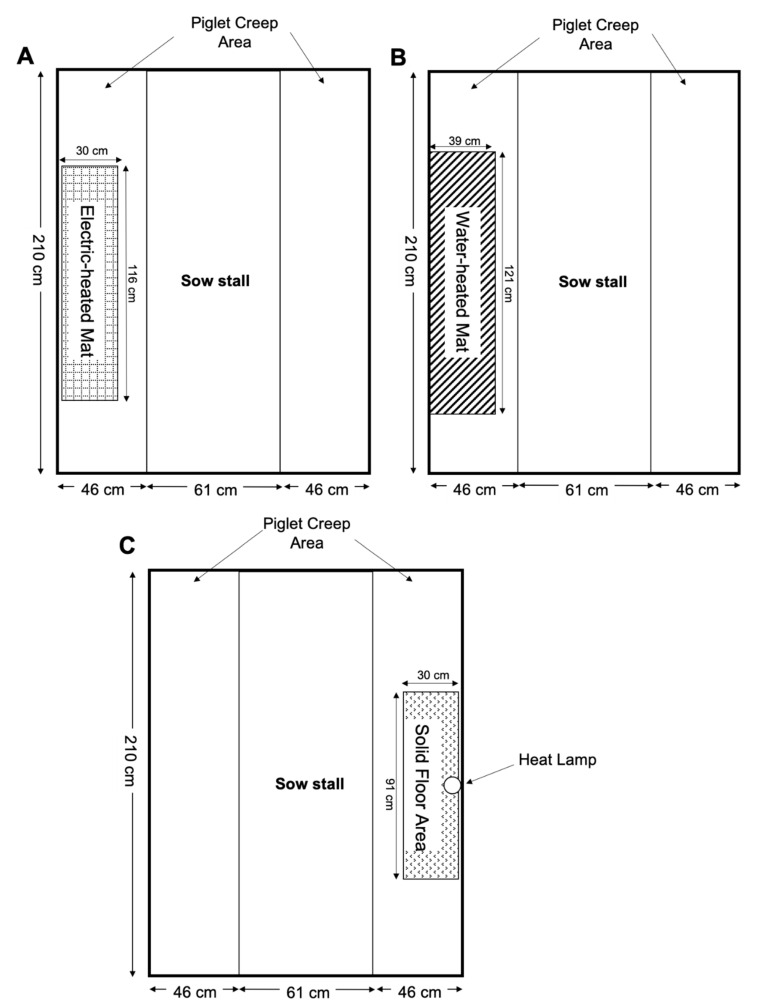
Layout of farrowing stalls: (**A**). Electric-heated mat; (**B**). water-heated mat; and (**C**). heat lamp.

Two days before the first expected farrowing date, 32 sows that were expected to farrow within a week of each other were transferred to two, mirrored confinement farrowing rooms (Room A and Room B). Both rooms were equipped with 16 individual farrowing stalls (150 cm × 210 cm) on slotted floors. Stalls within each room were arranged in two rows of eight stalls on either side of a central walkway. Sows faced the central walkway with a walkway behind them. Each stall contained a sow stall (61 cm × 210 cm) and a piglet creep area (46 cm × 10 cm) on each side of the sow stall (Figure 1). Flooring material under the sow was perforated cast iron with plastic-coated woven wire as flooring material in the piglet creep area. Each farrowing stall was equipped with a dry-feeder for the sow, and two nipple drinkers, one for the sow and one for the piglets. Stalls were mounted above a deep (2.4 m) anaerobic slurry collection pit. Farrowing room temperature was controlled at 20 °C, within the thermoneutral zone for lactating sows as much as possible by thermostats that operated exhaust fans and heaters. Light period was set at 8 h daily starting from 0700 h. 

All sows and piglets were managed according to the WCROC’s standard operating procedures for farrowing and lactation. Sows farrowed naturally without artificial induction. Beginning the day after parturition, sows were fed an increasing quantity of feed according to appetite until day 4 postpartum. After day 4 postpartum, sows were allowed ad libitum access to feed until the day of weaning. Within 24 h after birth, piglets were processed (e.g., surgical castration, docking tail, and iron injection) and cross-fostered if the litter size was larger than 14. Only piglets heavier than the mean weight within litter were cross-fostered to ensure their compatibility in the recipient litter. Piglets were not offered creep feed and were weaned at 19.4 ± 1.20 (SD) days of age. After weaning, sows were moved to the gestation barn for heat detection and mating for the next breeding cycle.

### 2.2. Heat Sources for Piglets 

Three supplemental heat sources for piglets, electric-heated mat (EM), water-heated mat (WM), and heat lamp (HL), were employed in this study. 

Electric-heated mats were installed in Room A. The EM (75 W, Model NH75, Innovative Heating Technologies) measured 30 cm × 116 cm and was secured in one creep area of each stall (Figure 1A). One pre-programmed heat mat controller (HMC, Hog Hearth^®^, Innovative Heating Technologies Inc., Oak Bluff, MB, Canada) was installed for controlling temperature of all EM in the room. Temperature of EM was set at 36 °C for the first 7 days postpartum then was reduced in a stepwise manner over the next 10 days to 31 °C. Temperature setting remained at 31 °C until piglets were weaned.

Water-heated mats were installed in Room B and secured in one creep area of each stall with a similar position to EM (Figure 1B). Water-heated mats were custom-made of stainless steel plate and measured 39 cm × 121 cm. These mats were single-walled with a central partition extending most of the length inside the mat to create a U-shaped channel. This configuration allowed hot water to be circulated through the mat to heat piglets by entering on one side of the partition and exiting on the other side. Sensors (Badger Meter Series 380 BTU system, Milwaukee, WI, USA) measured the overall supply (inlet) and return (outlet) water temperature in the WM system. The surface temperature of each WM was assumed to be similar to the temperature of water circulated through the mats. Inlet and outlet water temperatures were 35.8 °C and 33.8 °C, respectively for Trial 1 and 35.3 °C and 34.4 °C, respectively for Trial 3. Hot water from a liquid-to-liquid heat pump was circulated to WM and cooler water returned to the heat pump in a closed loop with a Tichelmann system configuration that ensured all WM received water of the same temperature for heating the mats. 

Heat lamps were installed when needed based on trial requirements in both Room A and Room B. Infrared heat lamps (125 W, Model S4750, SATCO Products Inc., Brentwood, NY, USA) equipped with an aluminum, heat-deflection shroud were used. Heat lamps were suspended (53 cm) above the floor in one creep area of each sow stall (Figure 1C). A solid floor area (30 cm × 91 cm) was located under each lamp to eliminate updrafts from the underfloor manure collection pit. An infrared thermometer (Performance Tool^®^, Model W89721, Renton, WA, USA) determined that each heat lamp provided a microenvironment temperature of 27.2 °C to 30.6 °C on the solid floor surface. Floors in the piglet creep area were plastic-coated expanded metal. 

### 2.3. Experimental Design 

To evaluate the effects of WM and EM on piglet behavior and performance, three trials were conducted. Each trial lasted 3 weeks. In Trials 1 and 2, we compared heat mats (WM or EM) to heat lamps. Heat lamps were considered the Control treatment because they are commonly used as a supplemental heat source for piglets in commercial farrowing barns. In Trial 3, we compared the two heat mat designs. 

Trial 1 was conducted in January and February of 2018. Room A was equipped with heat lamps and Room B was fitted with water-heated mats. Sixteen focal sows (Parity = 4.0 ± 0.26) were selected for data collection based on parity and expected farrowing date to minimize variation among sows. Among the focal sows, 8 sows were allocated to each room to balance parity and farrowing date between the two rooms. Within each room, focal sows were allocated randomly to non-adjoining stalls where video cameras were installed. Focal sows in both rooms farrowed within 4 days of each other. 

Trial 2 was conducted in March and April of 2019. In Room A, EM provided heating to piglets and HL provided supplemental heat to piglets in Room B. The WM installed in Room B were not operating during this trial. The number of focal sows (*n* = 16, parity = 2.3 ± 0.39) used for data collection, and selection and allocation of focal sows were similar to that described for Trial 1. Focal sows farrowed within 3 days of each other.

Trial 3 was conducted in June 2018. In Room A and Room B, EM and WM provided supplemental heat to piglets, respectively. The number of focal sows (*n* = 16, parity = 2.7 ± 0.47), and selection and allocation of focal sows were similar to that described for Trial 1. This trial was part of the large project that evaluated effects of a floor cooling pad on heat stress of lactating sows [17]. Consequently, room temperature was controlled to target 29.4 °C during daytime (between 0700 and 1900 h), and 23.9 °C during nighttime (between 1900 and 0700 h) from the day that sows were moved to the farrowing rooms until weaning [17]. Average daytime temperatures recorded for Rooms A and B were 29.4 and 28.4 °C, respectively, and nighttime temperatures were 26.6 and 25.9 °C, respectively.

### 2.4. Data Collection

#### 2.4.1. Behavior of Piglets

For all three trials, piglet behavior was recorded by infrared digital cameras and a computer equipped with video-recording software (Geo Vision Multicam Digital Surveillance System V8.2; USA Vision System Inc., Irvine, CA, USA). In each room, 8 cameras (TruVision High Definition TVI Bullet Camera TVB-4403, Interlogix, Costa Mesa, CA, USA) were mounted on the ceiling above 8 farrowing stalls. Video recording began one day before the earliest expected farrowing day of all focal sows in both rooms. Video recording continued 24 h daily until 7 days after birth of piglets by all focal sows. Piglet behavior was recorded again on about days 14 and 21 postpartum from 0000 h to 2400 h. 

Video recordings were viewed for postures and locations of piglets by trained observers using instantaneous scan-sampling at five-minute intervals [18]. During training sessions, observers were asked to collect data from the same video segments. Observers were considered ready for data collection when inter-rater agreement reached 90% or greater. For trial 1 and trial 3, video recordings were scan-sampled for 24 h on days 1, 3, 7, 14, and 21 after birth. For trial 2, data were collected for 24 h on day 1 and day 3 only due to technical difficulties in video-recordings. Postures of interest included lying and standing. Lying was defined as lying laterally or sternally, and standing was defined as standing up on four legs still or moving [19]. Sitting (front legs upright with hindquarters touching the floor) was rarely observed (less than 1% of time budget) so it was combined with lying. Locations of piglets were recorded as on the heat mat or solid floor area under HL, and outside the heat source. Piglets were considered to be on the heat mat or under the HL when at least half of its body (torso) was in that area. These postures and locations were mutually exclusive. Consequently, there were four combinations of postures and locations recorded: Lying on mat or solid floor under HL, standing on mat or solid floor area under HL, lying outside the mat or solid floor area under HL, and standing outside the mat or solid floor area under HL. At each scan, observers recorded the number of piglets in each posture and location combination. Behavioral time budget for each posture and location combination was defined as time spent on each behavior in a certain location as a percentage of the total observation time [18]. Time budget was calculated as the number of pigs in each posture/location combination as a percentage of total number of piglets in the farrowing stall over the entire scan sampling period. For example, if a behavior was exhibited by 60% of piglets during 40 out of 288 sampling points (24 h at five-minute intervals), the time budget would be calculated as: [0.60 × (40/288)] × 100 = 8.3%. To evaluate usage of supplemental heat source by piglets, total time spent lying or standing on the heat mat or under HL was recorded. In addition, the percentage of total heated area (the area of WM, EM, or the solid floor area under the HL) covered by piglets was estimated visually.

#### 2.4.2. Performance of Piglets

At birth, total number of piglets born, born alive, stillborn, and mummified were recorded for each litter. Live piglets were weighed within 24 h after birth and again at weaning. Number of piglets that were cross-fostered into or out of each litter, and litter size after cross-fostering were also recorded. Number of piglets that died from birth to weaning was recorded and pre-weaning mortality was calculated as percentage of live born piglets for each litter. Average piglet weight at birth and weaning was calculated by dividing the total litter weight by the number of piglets weighed. 

### 2.5. Data Analysis

While data were analyzed separately for each trial, the same statistical models were used for data analysis across the three trials. Glimmix Procedures of SAS 9.4 (Cary, NC, USA) [20] were used to test the difference in piglet behavior and performance in response to different heat source treatments. The Poisson, Gaussian, or negative binomial regression models were used to fit distribution of the data. Data that were not distributed normally were transformed (square root or logarithm) to achieve normal distribution. Specifically, data of time spent lying on heated surface, total time spent on heated surface, and proportion of heated surface covered by piglets were square root transformed, and data for time spent lying outside heated surface were logarithm transformed. 

Dependent variables included piglet performance and behavioral time budget. For piglet performance, the model included heat source treatment (EM, WM, or HL) as the fixed effect. For analysis of behavioral data, Glimmix Procedures with repeated measures were used with heat source treatment, day (day 1, 3, 7, 14, or 21) after birth, and their interaction as fixed effects. In all cases, litter was the experimental unit. Results are presented as least-square means with pooled standard errors (the maximum SE within the group). For transformed data, transformed least-square means are presented with raw means in parentheses. Differences among least-square means were tested using Tukey’s test adjusted for multiple comparisons. All tests were two-tailed tests. A *p*-value less than (or equal to) 0.05 was considered statistically significant; and a *p*-value greater than 0.05 and less than (or equal to) 0.10 was considered a tendency.

## 3. Results

### 3.1. Trial 1: Comparing the Effect of Water-Heated Mat and Heat Lamp on Piglet Performance and Behavior 

Due to technical difficulties with video-recording, data were only collected on litters born to 7 focal sows in WM treatment and 6 focal sows in HL treatment (Table 1). Litter size born alive and after cross-fostering showed no difference between treatments. The number of piglets that died before weaning tended to be higher (*p* = 0.053) in the WM treatment, which tended to increase pre-weaning mortality (*p* = 0.06) compared to the HL group. Providing WM tended to decrease weaning weight of litters (*p* = 0.10) and average daily litter weight gain (*p* = 0.054) compared to HL treatment. Treatments had no effect on average piglet weights or average daily piglet weight gain.

Piglets provided with a WM spent more time on the mat compared with piglets provided a HL (*p* = 0.002). Time spent lying on the mat was longer (*p* = 0.001) for the WM compared with the HL groups, but time spent standing on the mat or under the HL was not different between the two groups. Compared to HL, WM decreased time pigs spent lying off the mat (*p* = 0.001), and tended to lower time pigs spent standing away from the mat (*p* = 0.09). No difference was observed in heated area covered by piglets between the two treatments. 

Age (day after birth) affected time spent lying on the mat (*p* = 0.03; Table 1), total time spent on the mat (*p* = 0.01), and time spent lying away from the mat (*p* = 0.01). Age tended to affect time piglets spent standing on the mat (*p* = 0.07), but had no effect on time spent standing away from the mat or the heated area occupied by piglets. 

Piglets spent a longer period lying on the mat on day 3 than day 21 (*p* = 0.02; Table 2) and tended to spend more time than day 14 (*p* = 0.097). On day 21, piglets stood on the mat less than on day 7 (*p* = 0.01). Time spent on the mat was longer on day 3 compared to day 21 (*p* = 0.01). Piglets spent less time lying away from the mat on day 3 compared to days 1, 14, and 21 (*p* < 0.05). Time spent lying away from the mat on day 7 tended to be longer than on day 3 (*p* = 0.086) and shorter on day 21 (*p* = 0.053). 

**Figure 2:**
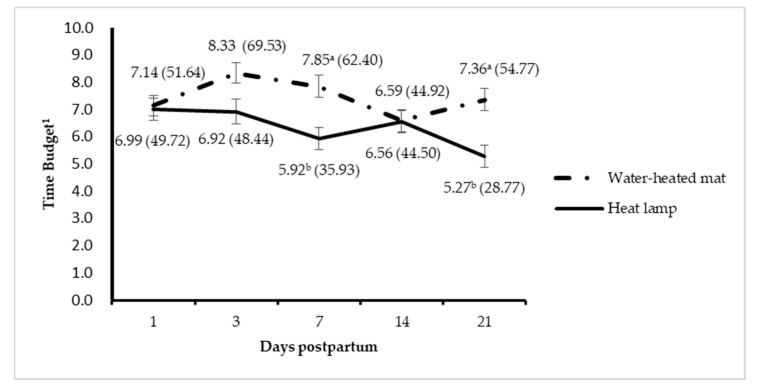
Time spent by piglets lying on heat mat or under the heat lamp over 21 days. ^1^ Time spent lying on water-heated mats or under heat lamps as a percentage of total observation time (24 h for each day). ^a,b^ Least square means within a day without a common superscript differ (*p* < 0.05).

**Figure 3:**
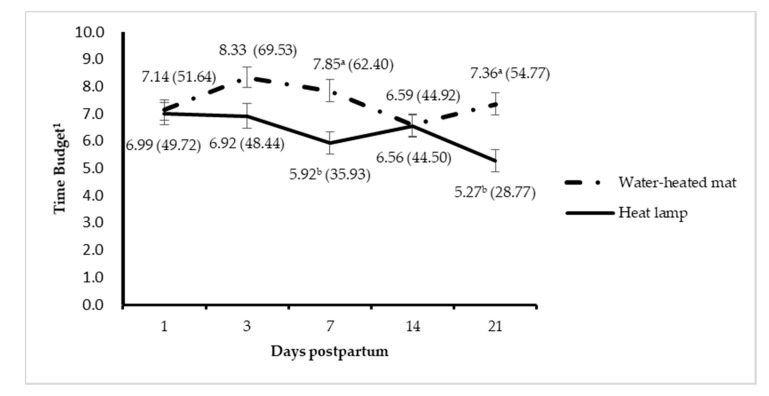
Time spent by piglets lying off the heat mat or away from the heat lamp over 21 days. ^1^ Time spent lying outside water-heated mats or heat lamps as a percentage of total observation time (24 h for each day). ^a,b^ Least square means within a day without a common superscript differ (*p* < 0.05).

We observed an interaction between treatment and day after birth on time spent lying on the mat (*p* = 0.03). Piglets in WM group spent more time lying on mat than piglets in HL group at day 7 (*p* = 0.002, Figure 2) and day 21 (*p* = 0.01) but not on other observation days. There tended to be an interaction between treatment and day on time spent lying away from the mat (Figure 3). Piglets spent less time lying off the WM than away from the HL at day 3 (*p* = 0.02), but not on other days. The time spent lying off the mat on day 3 was shorter compared to that on days 14 and 21 (*p* < 0.04).

### 3.2. Trial 2: Comparing the Effect of Electric-Heated Mat and Heat Lamp on Piglet Performance and Behavior 

Neither litter size, litter weight nor piglet growth rate were influenced by supplemental heat source (Table 3). Apart from increasing time budget for standing off the EM (*p* = 0.001), supplemental heat source did not affect piglet behavior. 

Time spent lying on the mat (*p* = 0.03; Table 4) and area covered by piglets (*p* = 0.002) were lower at day 1 compared to day 3. Total time spent on the mat (*p* = 0.058) and lying outside the mat (*p* = 0.063) tended to be lower while time spent lying outside the heat source tended to be greater on day 1 in contrast to day 3. No interaction between treatment and age was detected for any variable measured. 

### 3.3. Trial 3: Comparing the Effect of Water-Heated Mat and Electric-Heated Mat on Piglet Performance and Behavior 

In this trial, data were collected on 13 litters, 7 for the WM treatment and 6 for the EM treatment. Litter size born alive (*p* = 0.10) and weaned (*p* = 0.08) tended to be larger when WMs were provided compared to when EMs were provided (Table 5). No difference in litter size after cross-fostering or number of piglets dead per litter was observed between treatments. Piglets provided a WM tended to have a lower average individual weight (*p* = 0.07) at birth, though no difference was detected in litter birth weight between WM and EM treatment groups. Litter weight and individual pig weight at weaning were not different between WM and EM treatments. Average daily litter weight gain tended (*p* = 0.09) to be greater for litters offered the WM compared with litters provided the EM but there were no statistical differences in daily weight gain of individual piglets. Piglets spent more time lying on the WM (*p* = 0.01) which contributed to the increased total time spent on the WM compared with the EM (*p* = 0.02). The effect of supplemental heat source on time spent standing on mats, lying away from mats, and standing away from mats was not significant. A greater percentage (*p* = 0.02) of the WM was covered by piglets compared with the EM.

Age affected all postural behaviors (*p* < 0.01) and tended (*p* = 0.08) to influence the proportion of the mat covered by piglets. As age increased, piglets spent less time lying and standing on mats (Table 6). Time spent lying on the mat and total time spent on the mat decreased until day 7 (*p* < 0.001) and remained constant thereafter. Time spent standing on mats decreased as age increased from days 1 to 14 (*p* = 0.02). Piglets dedicated more time to lying off mats as age increased from day 1 to day 7 (*p* < 0.001). Time spent standing off the mat decreased until day 3 (*p* = 0.002). Age did not affect the proportion of the mat area covered by piglets. No interaction between treatment and age was detected for any variable measured. 

## 4. Discussion

Newborn piglets possess a limited capability of regulating internal body temperature in the first days of life [1]. Providing localized, supplemental heat to newborn piglets is a critical management practice in swine farrowing operations. Heat can be provided using equipment of varying design and powered with differing energy sources. When choosing a supplemental heat source, several considerations need to be taken into account such as: (1) Thermal efficiency, (2) efficacy of heating piglets, (3) cost and ease of maintenance and operation, and (4) cost and ease of installation. Here, we chose three types of supplemental heat (infrared heat lamp, electric-heated mat, and water-heated mat) to evaluate. 

Heat lamps have been the standard supplemental heat source for piglets in commercial swine production units for years [10]. Heat lamps are easy to install and use. Initial cost of heat lamps is low but useful life is relatively short because they are easily broken especially when farrowing rooms are cleaned and heat lamps are moved routinely. Furthermore, energy efficiency is low for heat lamps compared to other sources of supplemental heat which increases cost of operation [13]. Heat provided by lamps is radiant in a top-down pattern which heats standing or moving piglets better than lying piglets. In addition, heat from a lamp is concentrated right under the bulb with decreased intensity as one moves to the periphery of the heated area. This spatial variation of heat could provide a choice for piglets to select the area with a temperature they prefer. Conversely, the temperature might be much warmer than desired right under the bulb (hot spot) and insufficient to satisfy the piglets’ thermal need at the periphery. This spatial variation in temperature often results in limited usable heated region and regions that might even repel piglets from using the heated area [11,21]. Heat from lamps can contribute to heat stress of the sow with concurrent sow discomfort which can contribute to additional crushing deaths of piglets [12]. Despite their drawbacks, heat lamps have proved effective in reducing hypothermia and mortality of piglets [12]. 

Compared to heat lamps, heat mats generally provide a more evenly distributed surface temperature, with variable sizes for installation in farrowing stalls [13]. Heat provided by mats is conducted through the surface of the mat. Bottom-up heating is considered to be more economical and practical than top-down heating during the farrowing and nursery periods [22]. During these stages of production, pigs spend more time lying than standing [23]. Bottom-up heating warms piglets through their stomachs and can be utilized with minimal waste. The heat mats studied have two types of heat inputs: embedded electric elements and circulated hot water. 

Electric-heated mats are easy to use once electrical receptacles and the mat controller have been installed. Compared to heat lamps, electric heat mats have a higher initial cost but are more energy efficient [24]. Previously, researchers calculated that electricity savings realized using electric heated mats compared with heat lamps for one year would equal the cost of installing the electric mats [10]. Using our costs for electric-heated mats, electricity savings compared with heat lamps could cover the initial purchase and installation costs of the electric-heated mats in about 5 years of operation. Unlike heat lamps that typically are replaced at least once per year or more frequently, EMs provide years of useful life. Therefore, EMs are preferable to HL when considering operational costs alone. But, when one considers the higher initial investment cost of electric-heated mats combined with the electricity savings compared with heat lamps, the economic decision to use electric-heated mats is less clear. Efficacy of heat mats to enhance piglet well-being and productivity are not well-explained. In many cases, temperature of occupied mat regions could be markedly higher than that of unoccupied regions which challenges the ability of the temperature sensors to maintain a consistent mat temperature [24]. Newborn piglets [18] and piglets weighing less than 1.7 kg [22] preferred heat lamps to heat mats. However, this preference shifted to heat mats as body weight of pigs increased [22]. We could not evaluate piglet preferences in the current study because only one supplemental heat source was offered to piglets within each treatment group. In the present study, we found no differences in piglet growth rate or usage of the heat source by piglets when comparing HL and EM (Trial 2). The comparable effect of HL and EM on piglet growth performance and behavior has been reported by other researchers [10,21,25]. Some investigators [18] suggested that piglets began use of HL about one day earlier than EM, but no interaction between age and supplemental heat source (HL or EM) was observed in the present study.

Compared to EM and HL, WMs are complicated and expensive to install and potentially have greater variation of surface temperature across the heated zone. Variation in surface temperature of the WM occurs when air bubbles are trapped in the mat which limits heat transfer from the circulating hot water to the mat surface. There is a paucity of scientific literature reporting the feasibility of WM in swine farrowing operations. In the present study, HL supported faster litter growth rate and lower piglet mortality compared with WM (Trial 1). Pre-weaning growth and mortality rates are regulated by complex interactions among piglet factors (birth weight, colostrum intake, thermoregulatory ability, physical strength, and gender), sow factors (colostrum production, parity, and maternal characteristics), and environmental factors (temperature, housing, and management) [26]. We suspect WM did not adequately satisfy piglets’ thermal needs which resulted in poorer growth performance and increased mortality compared with piglets offered HL. This suspicion is supported by the observation that piglets spent significantly more time on WM than under HL presumably seeking increased warmth. 

The difference in mortality rate and weight gain between WM and HL might also be attributed to the small differences in litter size and individual piglet birth weight. Piglets provided WM were born slightly lighter than pigs provided HL (1.2 vs. 1.5 kg) but this difference was not statistically significant. Several researchers [26,27,28] have reported an inverse relationship between birth weight and pre-weaning mortality of suckling pigs. Clearly, supplemental heating treatments had no effect on litter size farrowed or birth weight of piglets. The increased mortality rate of piglets assigned to WM likely resulted from a combination of the random occurrence of lighter piglet birth weight and marginally effective heating provided by the WM. 

The comparison between WM and EM was conducted as part of a larger study in which sows were subjected to heat stress conditions (29.4 °C for 0700–1900, 23.9 °C for 1900 to 0700; Trial 3). In WM group, average daily litter weight gain tended to be greater but pre-weaning piglet mortality was similar compared to EM. Clearly, there was less need for supplemental heat for piglets in this trial because piglets spent 60% less time in the heated zone compared with Trials 1 and 2. Similarly, the proportion of the heated zone covered by piglets was about 20% in Trial 3 compared with about 45 to 50% in Trials 1 and 2, respectively. 

Litters assigned to WM tended by random chance to be larger than those assigned to EM which resulted in a tendency for lighter individual piglet birth weight for WM litters [29]. The observed tendency for increased litter growth rate with WM likely was the result of larger litters because growth rate of individual piglets was not different between WM and EM. Piglets with lower birth weight normally have less developed thermoregulation capabilities compared with heavier birth weight littermates [5]. Thus, the need for supplemental heat likely was greater for piglets provided WM than those offered EM which explains the higher usage and coverage of the mat in the WM compared with EM groups. 

In trials 1 and 2, we observed a tendency for increased usage of the heat source (as measured by total time spent in the heated area) by piglets on day 3 compared to day 1, then usage gradually decreased as piglets aged. This pattern of heat source usage has been reported in previous studies [30,31,32]. Piglets naturally prefer to stay close to the sow during the first 1 to 2 days after birth, and will most commonly begin using heat sources after day 2 of age. As piglets grow, their thermoregulatory capabilities mature and the need for supplemental heat declines. However, under heat stress conditions for the sow (Trial 3), usage of heat source was decreased and time spent outside the heated area was increased from day 1 of age onward. We suggest this was because of a reduction in piglets’ thermal need at a room temperature of 29.4 °C [7]. Interactions between treatment and age on behavioral time budget were not consistent across the three trials. 

Heated mats provide a solid floor surface for piglets to lay on which could become soiled with feces and urine in some situations. Soiled mats likely would provide uneven heat distribution and unsanitary conditions for suckling piglets. Unsanitary conditions might require additional labor for cleaning the floor surface that would not be required for piglets under heat lamps on perforated floors. While this situation may occur on farms, we observed no soiling of heat mats in any of the three trials conducted. 

We acknowledge that the heated area provided by HL, EM, and WM was not uniform across treatments. The WM provided the largest heated area, followed by the EM and then the HL. Using average birth weight observed in our trials (1.5 kg) and the equation to estimate floor space needs from body weight (A (m^2^) = 0.025 BW^0.66^ (kg)) provided by Petherick and Clive [33], we estimate that the WM, EM, and HL could accommodate 14.4, 10.6, and 8.3 pigs lying, respectively. These differences potentially influenced piglets’ time budget for use of the heat source and proportion of heated area covered by piglets. However, heated area occupied by piglets in all three trials never reached 100% which suggests that the total area heated by the supplemental heat source did not limit use of the heat source by piglets. 

## 5. Conclusions

In conclusion, HL and EM were comparable with regard to pre-weaning survival and growth rate of piglets and piglet use of the heating source. The WM used in this experiment did not consistently improve piglet performance compared with HL or EM and are more difficult and costly to install in commercial settings. Considering the ease of installation, piglet growth performance and survival, and the greater energy efficiency, EM appear to be the most effective choice to provide supplemental heat to suckling piglets.

## Figures and Tables

**Table 1:** Behavior and performance of piglets using water-heated mats or heat lamps (Trial 1).

Item	Treatment		Pooled SE	*p*-Value		
	Water-Heated Mat	Heat Lamp		Treatment	Day	Interaction
# of sows	7	6	-	-	-	-
Sow Parity	3.7	4.5	0.43	0.20	-	-
Litter size, piglets/litter						
Born alive	14.7	13.9	1.22	0.63	-	-
After cross-fostering ^1^	14.1	13.4	0.42	0.18	-	-
Dead ^2^	3.3	1.1	0.94	0.053	-	-
At weaning ^3^	10.8	12.3	0.81	0.23	-	-
Mortality ^4^,%	22.9	8.9	7.63	0.06	-	-
Litter weight, kg						
At birth ^5^	18.1	19.5	1.63	0.56	-	-
At weaning ^3^	60.5	73.5	5.20	0.10	-	-
Average piglet weight, kg						
At birth ^5^	1.2	1.5	0.10	0.11	-	-
At weaning ^3^	5.5	6.1	0.30	0.22	-	-
Average daily litter gain ^6^, kg	2.4	3.0	0.19	0.054	-	-
Average daily piglet gain ^6^, g	224.7	242.9	10.40	0.21	-	-
Behavioral time budget ^7^,%						
Lying on mats or under heat lamps	7.5 (56.0) ^8^	6.3 (40.6)	0.19	0.001	0.03	0.03
Standing on mats or under heat lamps	3.3 (11.3)	3.1 (10.0)	0.14	0.28	0.07	0.81
Total time spent on mats or under heat lamps	8.2 (67.5)	7.1 (51.0)	0.19	0.002	0.01	0.11
Lying away from mats or heat lamps	2.9 (10.4)	4.7 (24.7)	0.33	0.001	0.01	0.10
Standing away from mats or heat lamps	4.3 (19.0)	4.7 (22.3)	0.17	0.09	0.11	0.12
% of total mat area covered by piglets	6.6 (43.7)	6.9 (48.9)	0.18	0.23	0.19	0.05

^1^ Cross-fostering was conducted within 24 h after birth to achieve litter size of 14 or less as much as possible. ^2^ Number of piglets that died from birth to weaning. ^3^ Piglets were weaned at 21 day of age. ^4^ Percent of piglets born alive that died from birth to weaning (number of piglets that died/born alive × 100%). ^5^ Piglets were weighed within 24 h after birth. ^6^ Calculated by weight difference between birth and weaning divided by age at weaning. ^7^ Time spent as a percentage of total observation time (24 h for each day). For location identification, at least one half of the piglet’s body needed to occupy a location to be recorded. ^8^ Least square means (LSM) of raw means not distributed normally are displayed as square root or logarithm transformed LSM with raw LSM presented in parentheses. SE = Standard Error.

**Table 2:** Selected time budget for postures at different days of age (Trial 1).

Item	Day after Birth					Pooled SE
	1	3	7	14	21	
Behavioral time budget ^1^, %						
Lying on mats or under heat lamps	7.1 ^ab^ (50.7) ^2^	7.6 ^ae^ (58.0)	6.9 ^ab^ (39.7)	6.6 ^abf^ (44.7)	6.3 ^b^ (39.7)	0.2922
Standing on mats or under heat lamps	3.4 ^ab^ (12.3)	3.4 ^ab^ (11.7)	3.5 ^a^ (13.7)	3.0 ^ab^ (9.6)	2.7 ^b^ (7.3)	0.2248
Total time spent on mats or under heat lamps	7.9 ^ab^ (62.9)	8.4 ^a^ (69.8)	7.8 ^ab^ (61.9)	7.3 ^ab^ (54.4)	6.9 ^b^ (41.1)	0.3035
Lying away from mats or heat lamps	3.7 ^ae^ (16.5)	2.4 ^bg^ (7.6)	3.5 ^abf^ (16.4)	4.5 ^a^ (20.8)	4.9 ^ae^ (24.8)	0.5272

^1^ Time spent as a percentage of total observation time (24 h for each day). For location identification, at least one half of the piglet’s body needed to occupy a location to be recorded. SE = Standard Error. ^2^ Least square means (LSM) of raw means not distributed normally are displayed as square root or logarithm transformed LSM with raw LSM presented in parentheses. ^a,b^ Means within a row without a common superscript differ (*p* < 0.05). ^e,f,g^ Means within a row without a common superscript tend to differ (0.05 < *p* < 0.10). SE = Standard Error.

**Table 3:** Behavior and performance of piglets using electric-heated mats or heat lamps (Trial 2).

Item	Treatment		Pooled SE	*p*-Value		
	Electric-Heated mat	Heat Lamp		Treatment	Day	Interaction
# of sows	8	8	-	-	-	-
Sow Parity	2.9	2.5	0.59	0.65	-	-
Litter size, piglets/litter						
Born alive	13.6	13.3	1.47	0.86	-	-
After cross-fostering ^1^	13.6	12.9	0.88	0.55	-	-
Dead ^2^	1.6	1.8	0.81	0.91	-	-
At weaning ^3^	12.0	11.1	0.67	0.37	-	-
Mortality ^4^, %	10.7	9.9	4.73	0.90	-	-
Litter weight, kg						
At birth ^5^	20.0	19.8	1.71	0.92	-	-
At weaning ^3^	77.0	75.3	4.97	0.81	-	-
Average piglet weight, kg						
At birth ^5^	1.5	1.6	0.11	0.61	-	-
At weaning ^3^	6.5	6.8	0.29	0.48	-	-
Average daily litter gain ^6^, kg	3.1	2.9	0.20	0.53	-	-
Average daily piglet gain ^6^, g	258.2	261.1	13.05	0.88	-	-
Behavioral time budget ^7^,%						
Lying on mats or under heat lamps	7.1 (50.5) ^8^	7.0 (49.3)	0.26	0.80	0.03	0.25
Standing on mats or under heat lamps	2.1 (4.7)	2.2 (5.1)	0.17	0.74	0.41	0.34
Total time spent on mats or under heat lamps	7.4 (55.2)	7.3 (54.6)	0.26	0.86	0.058	0.19
Lying away from mats or heat lamps	5.8 (34.5)	6.3 (40.9)	0.33	0.28	0.06	0.15
Standing away from mats or heat lamps	8.4	4.5	0.76	0.001	0.93	0.63
% of total mat area covered by piglets	7.3 (53.4)	7.2 (52.9)	0.24	0.89	0.002	0.28

^1^ Cross-fostering was conducted within 24 h after birth to achieve litter size of 14 or less as much as possible. ^2^ Number of piglets that died from birth to weaning. ^3^ Piglets were weaned at 21 day of age. ^4^ Percent of piglets born alive that died from birth to weaning (number of piglets that died/born alive × 100%). ^5^ Piglets were weighed within 24 h after birth. ^6^ Calculated by weight difference between birth and weaning divided by age at weaning. ^7^ Time spent as a percentage of total observation time (24 h for each day). For location identification, at least one half of the piglet’s body needed to occupy a location to be recorded. ^8^ Least square means (LSM) of raw means not distributed normally are displayed as square root or logarithm transformed LSM with raw LSM presented in parentheses. SE = Standard Error.

**Table 4:** Selected time budget for postures at different days of age (Trial 2).

Item	Days after Birth		Pooled	*p*-Value
	1	3	SE	
Behavioral time budget ^1^, %				
Lying on mats or under heat lamps	6.6 (44.3) ^2^	7.4 (56.1)	0.26	0.032
Total time spent on mats or under heat lamps	7.0 (49.9)	7.7 (60.5)	0.26	0.058
Lying away from mats or heat lamps	6.5 (43.6)	5.6 (32.4)	0.33	0.063
% of total mat area covered by piglets	6.6 (44.9)	7.9 (62.9)	0.24	0.002

^1^ Time spent as a percentage of total observation time (24 h for each day). For location identification, at least one half of the piglet’s body needed to occupy a location to be recorded. SE = Standard Error. ^2^ Least square means (LSM) of raw means not distributed normally are displayed as square root or logarithm transformed LSM with raw LSM presented in parentheses.

**Table 5:** Behavior and performance of piglets using water-heated mats or electric-heated mats (Trial 3).

Item	Treatment		Pooled SE	*p*-Value		
	Water-Heated Mat	Electric-Heated Mat		Treatment	Day	Interaction
# of sows	7	6	-	-	-	-
Sow Parity	3.0	2.9	0.71	0.90	-	-
Litter size, piglets/litter						
Born alive	15.1	12.6	1.10	0.10	-	-
After cross-fostering ^1^	14.4	12.6	1.04	0.21	-	-
Dead ^2^	1.3	1.5	0.52	0.77	-	-
At weaning ^3^	13.1	11.1	0.79	0.08	-	-
Mortality ^4^, %	8.1	10.9	3.58	0.54	-	-
Litter weight, kg	-	-	-	-	-	-
At birth ^5^	21.1	20.9	1.01	0.85	-	-
At weaning ^3^	69.8	63.5	3.87	0.25	-	-
Average piglet weight, kg						
At birth ^5^	1.4	1.7	0.10	0.07	-	-
At weaning ^3^	5.3	5.8	0.24	0.20	-	-
Average daily litter gain ^6^, kg	2.6	2.3	0.13	0.09	-	-
Average daily piglet gain ^6^, g	202.4	211.1	11.64	0.60	-	-
Behavioral time budget ^7^, %						
Lying on mats	3.6 (13.3) ^8^	2.9 (8.5)	0.15	0.01	<0.0001	0.96
Standing on mats	8.30	8.30	0.60	0.95	0.001	0.67
Total time spent on mats	4.7 (21.8)	4.1 (17.1)	0.14	0.02	<0.0001	0.87
Lying away from mats	59.0	62.0	1.33	0.11	<0.0001	0.98
Standing away from mats	17.9	19.1	0.71	0.20	0.003	0.89
% of total mat area covered by piglets	4.7 (23.0)	4.1 (17.2)	0.19	0.02	0.08	0.96

^1^ Cross-fostering was conducted within 24 h after birth to achieve litter size of 14 or less as much as possible. ^2^ Number of piglets that died from birth to weaning. ^3^ Piglets were weaned at 21 day of age. ^4^ Percent of piglets born alive that died from birth to weaning (number of piglets that died/born alive × 100%). ^5^ Piglets were weighed within 24 h after birth. ^6^ Calculated by weight difference between birth and weaning divided by age at weaning. ^7^ Time spent as a percentage of total observation time (24 h for each day). For location identification, at least one half of the piglet’s body needed to occupy a location to be recorded. ^8^ Least square means (LSM) of raw means not distributed normally are displayed as square root or logarithm transformed LSM with raw LSM presented in parentheses. SE = Standard Error.

**Table 6:** Selected time budget for postures at different days of age (Trial 3).

Item	Days after Birth					Pooled
	1	3	7	14	21	SE
Behavioral time budget ^1^, %						
Lying on mats	4.25 ^a^ (18.46) ^2^	3.89 ^a^ (15.82)	2.47 ^b^ (6.23)	2.89 ^b^ (8.57)	2.89 ^b^ (8.72)	0.23
Standing on mats	11.79 ^a^	9.24 ^abe^	8.39 ^ab^	7.22 ^b^	6.00 ^bf^	1.01
Total time spent on mats ^2^	5.47 ^a^ (30.35)	4.96 ^a^ (25.26)	3.85 ^b^ (14.87)	3.96 ^b^ (15.84)	3.78 ^b^ (14.77)	0.21
Lying away from mats	47.42 ^c^	58.29 ^b^	67.62 ^a^	65.2 ^a^	66.54 ^a^	2.09
Standing away from mats	21.14 ^a^	16.37 ^b^	17.22 ^b^	18.60 ^b^	18.58 ^b^	1.05
% of total mat area covered by piglets	4.79 ^e^ (23.40)	4.75 ^ef^ (18.37)	3.79 ^f^ (21.28)	4.20 ^ef^ (18.37)	4.46 ^ef^ (21.28)	0.28

^1^ Time spent as a percentage of total observation time (24 h for each day). For location identification, at least one half of the piglet’s body needed to occupy a location to be recorded. SE = Standard Error. ^2^ Least square means (LSM) of raw means not distributed normally are displayed as square root or logarithm transformed LSM with raw LSM presented in parentheses. ^a,b,c^ Means within a row without a common superscript differ (*p* < 0.05). ^e,f^ Means within a row without a common superscript tend to differ (0.05 < *p* < 0.10).
